# Extensive Mastectomy Skin Flap Necrosis Triggered by Hypertension, Psychological Stress, and Prolonged Operative Time Due to Intraoperative Needle Loss: A Case Report

**DOI:** 10.7759/cureus.95705

**Published:** 2025-10-29

**Authors:** Yudai Kaneda, Kenji Gonda, Akihiko Ozaki, Toyoaki Sawano, Takuya Oku, Tomohiro Kurokawa, Kazunoshin Tachibana, Masahiro Wada, Yoshiaki Kanemoto, Hiroaki Shimmura

**Affiliations:** 1 Clinical Training Center, Jyoban Hospital of Tokiwa Foundation, Iwaki, JPN; 2 Department of Breast Surgery, Jyoban Hospital of Tokiwa Foundation, Iwaki, JPN; 3 Department of Breast and Thyroid Surgery, Jyoban Hospital of Tokiwa Founation, Iwaki, JPN; 4 Department of Surgery, Jyoban Hospital of Tokiwa Foundation, Iwaki, JPN; 5 Department of Surgery, Yokohama Asahi Central General Hospital, Yokohama, JPN; 6 Department of Breast Surgery, Fukushima Medical University, Fukushima, JPN; 7 Breast and Thyroid Center, Jyoban Hospital of Tokiwa Foundation, Fukushima, JPN; 8 Department of Surgery, Jyoban Hospital, Iwaki, JPN; 9 Department of Urology, Jyoban Hospital, Iwaki, JPN

**Keywords:** breast cancer, hypertension, mastectomy skin flap necrosis, operative time, preoperative anxiety

## Abstract

Mastectomy skin flap necrosis (MSFN) is a frequent complication influenced by multiple factors. We report a 73-year-old woman with untreated hypertension and preoperative anxiety who developed extensive MSFN after mastectomy. Intraoperative loss of a suture needle prolonged operative time by approximately one hour, contributing to progressive flap necrosis requiring repeated debridement and negative pressure wound therapy. This case was documented to highlight the importance of recognizing how overlooked comorbidities, psychological stress, and intraoperative incidents can interact to exacerbate postoperative complications such as MSFN.

## Introduction

Breast cancer is the most frequently diagnosed malignancy among women worldwide, with approximately 2.3 million new cases and 670,000 deaths reported in 2022 [1]. For early-stage breast cancer, standard treatment consists of either breast-conserving surgery with adjuvant radiotherapy or mastectomy, depending on tumor characteristics and patient preference. Surgical intervention thus remains a cornerstone of breast cancer management.

However, mastectomy skin flap necrosis (MSFN) is a well-recognized postoperative complication, with reported incidence ranging from approximately 5% to as high as 30% [2]. Multiple risk factors have been identified, including advanced age, obesity, diabetes, smoking, prior irradiation, hypertension, and large breast size [2]. Among these, hypertension warrants particular attention as an independent risk factor that may impair microcirculation and wound healing at the surgical site [3]. Also, prolonged operative time is considered a potential risk factor for MSFN, as it may contribute to hypothermia and blood pressure fluctuations caused by prolonged anesthesia, as well as flap desiccation, microcirculatory impairment, and extended tissue ischemia [4]. Supporting this, one study in breast reconstruction found that the necrosis group had a significantly longer operative time than the non-necrosis group (260 vs. 229 minutes; p = 0.03) [5]. Moreover, increasing attention has been directed toward the impact of psychological stress and anxiety on postoperative wound healing [6]. Specifically, psychological stress, through cortisol release and sympathetic activation, disrupts immune and cytokine balance, impairs collagen synthesis and tissue remodeling, and ultimately delays wound healing while increasing the risk of necrosis [7-9].

When MSFN occurs, it can lead to surgical site infection, delayed wound healing, and the need for reoperation such as debridement or resuturing. These consequences may, in turn, delay the initiation of adjuvant therapy, worsen cosmetic outcomes, and ultimately diminish patients’ quality of life [2]. Nevertheless, evidence integrating operative time management and psychological factors in the prevention of MSFN following mastectomy alone remains limited. Here, we report the case of a 73-year-old woman with a history of hypertension who exhibited marked preoperative anxiety. During mastectomy for ductal carcinoma in situ (DCIS), the procedure was temporarily interrupted due to inadvertent loss of a suture needle, necessitating an extended search and resulting in prolonged operative time. Postoperatively, the patient developed extensive MSFN.

## Case presentation

In May 2025, a 73-year-old Japanese woman with a past medical history of hypertension, but no prior breast cancer screening, presented to our hospital with a chief complaint of a right breast mass. She also reported associated skin erosion and erythema. She had no known drug allergies. Her family history was notable for gastric cancer in her mother. She lived with her husband and second daughter, had no history of smoking, but did consume alcohol. Although she had previously been diagnosed with hypertension at another hospital and prescribed antihypertensive medication, she had independently discontinued treatment, believing herself to be cured. As a result, she did not disclose her hypertension at the initial consultation, leading to its oversight.

**Figure 1:**
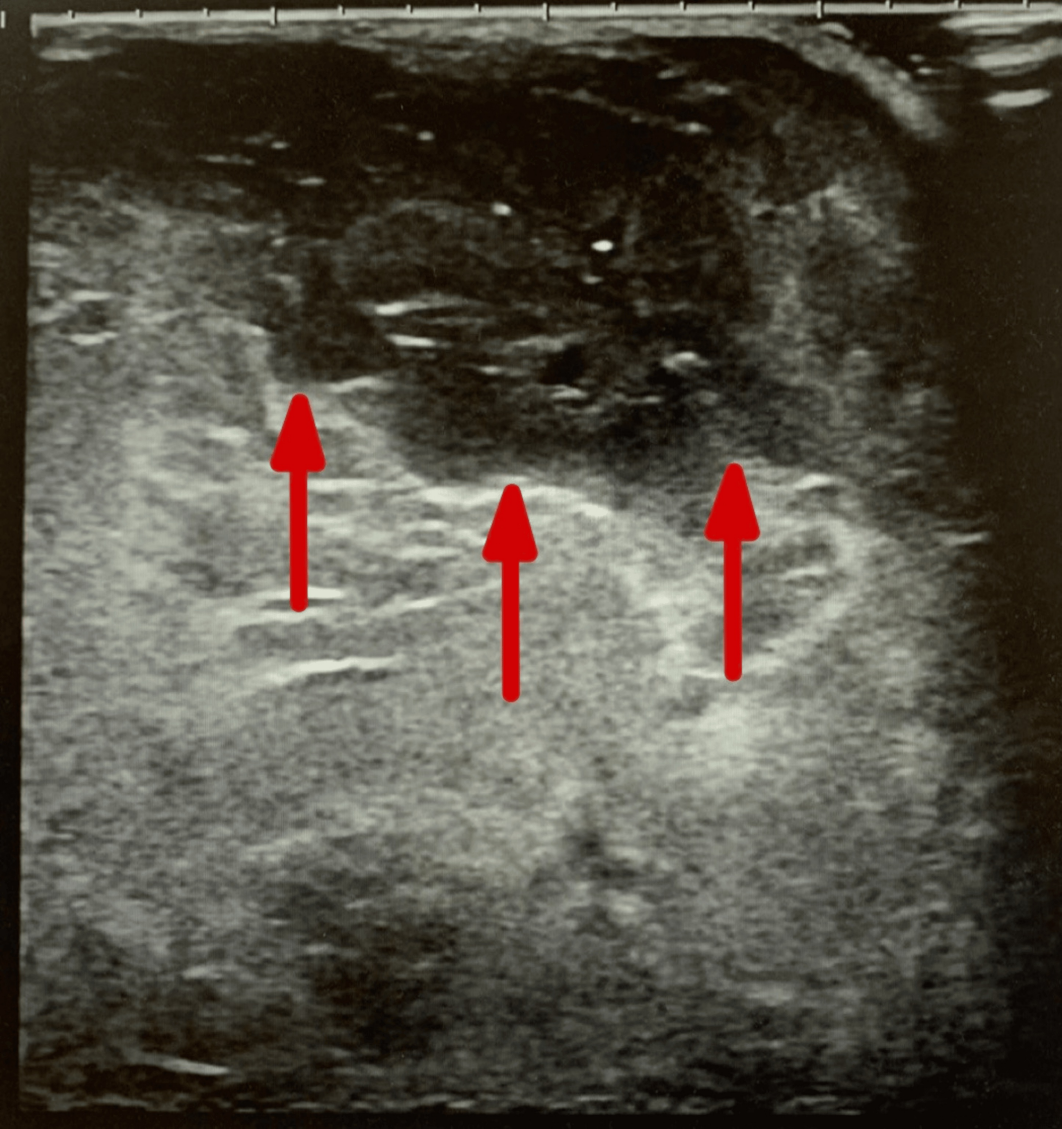
Preoperative ultrasonography of the patient

On physical examination, a mass approximately 10 cm in size was palpable in the upper outer quadrant of the right breast, accompanied by skin thickening, erythema, erosion, induration, and mild nipple retraction. Mammography revealed a high-density mass in the upper outer quadrant involving the nipple of the right breast, with parenchymal changes involving the nipple, and was classified as Breast Imaging Reporting and Data System (BI-RADS) category 5. Ultrasonography demonstrated a heterogeneous mixed solid mass measuring 99.2 × 71.7 × 87.4 mm in the upper and central regions of the right breast, with partially well-defined margins, internal heterogeneity, posterior acoustic enhancement, and punctate hyperechoic foci (Figure 1).

**Figure 2:**
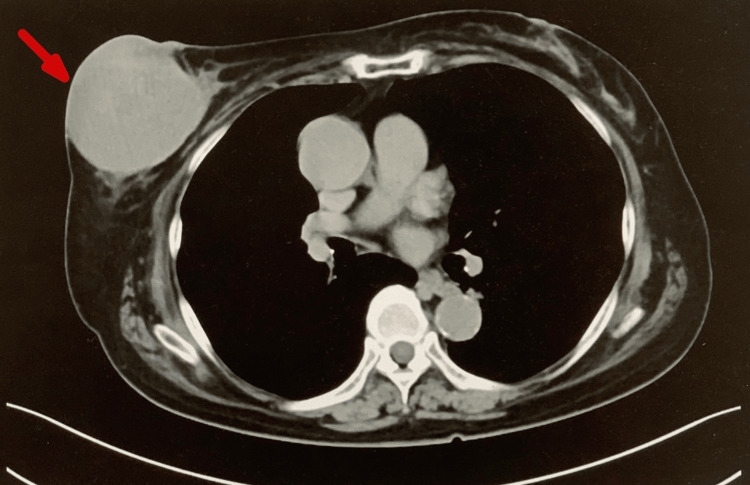
Preoperative computed tomography of the patient

Computed tomography (CT) revealed a cystic lesion measuring 73 mm in the central to lower outer quadrant of the right breast, as well as mild enlargement of the right axillary lymph nodes (Figure 2).

Positron emission tomography (PET) showed no evidence of distant metastasis. Based on these findings, the patient was diagnosed with right breast cancer, cTisN0M0, consistent with DCIS, Stage 0. Immunohistochemical analysis demonstrated estrogen receptor (ER) positivity, progesterone receptor (PR) positivity, HER2 negativity, and a Ki-67 proliferation index of 75%.

During preoperative admission on the day before surgery, the patient complained of anxiety, and her blood pressure rose to approximately 200 mmHg, which was managed with intravenous nicardipine. At this point, it was first revealed that she had previously been diagnosed with hypertension. On the day of surgery, her blood pressure stabilized at around 140 mmHg, and the procedure proceeded uneventfully according to the standard protocol. However, at the time of wound closure, a 4-0 polypropylene suture needle became dislodged from the instrument and was lost, prompting immediate interruption of the surgery for retrieval. Direct visual inspection failed to identify the needle, and two-view intraoperative radiography with a portable X-ray device was performed, but no radiopaque foreign body was detected within the operative field. It was therefore judged highly likely that the needle had fallen outside the body. During this period, the surgical wound was left open, anesthesia was maintained, and additional time was required to provide an explanation to the family. Approximately one hour later, wound closure was resumed, with two closed suction drains placed. The total operative time ultimately extended to three hours and 24 minutes, considerably longer than the typical duration of less than two hours [10]. The missing needle was eventually discovered during postoperative cleanup of the operating room.

**Figure 3:**
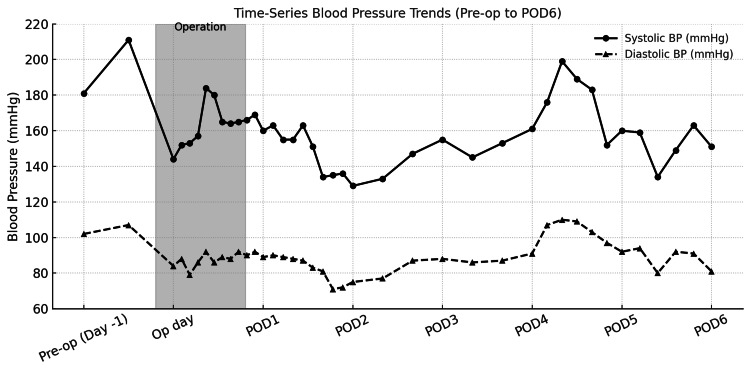
Time-Series Blood Pressure (BP) Trends (Pre-op to postoperative day (POD) 6)

Postoperatively, although no wound bleeding was observed, the patient’s blood pressure increased to the 160 mmHg range, requiring reinstitution of intravenous nicardipine. By the following day, her blood pressure had decreased; however, impaired perfusion of the upper portion of the skin flap was noted, and topical application of oxaceprol ointment (Exalb®) was initiated from postoperative day (POD) 2. On POD 5, the patient expressed a strong desire for early discharge, stating that prolonged hospitalization would increase her anxiety and consequently elevate her blood pressure. The surgical drains were removed, and she was discharged on POD 6. The daily changes in blood pressure from the day before surgery to POD 6 are shown in Figure 3.

**Figure 4:**
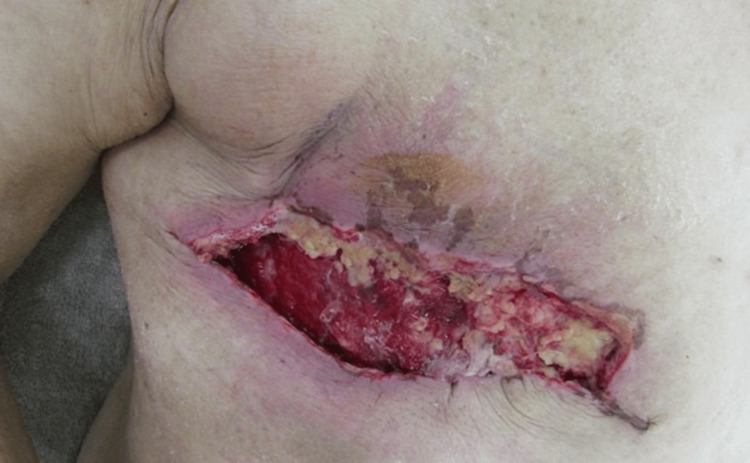
Progressive mastectomy skin flap necrosis at postoperative day 14

At the outpatient follow-up on POD 14, laboratory tests revealed leukocytosis (WBC 10,300/µL) and elevated C-reactive protein (CRP 3.83 mg/dL), suggestive of infection. Repeated wound debridement was performed in combination with systemic antibiotics and topical application of povidone-iodine sugar paste. Despite these interventions, the necrosis of the surgical site progressed to a massive extent (Figure 4). 

Repeated wound debridement was performed in combination with systemic antibiotics and topical application of povidone-iodine sugar paste. Despite these interventions, the surgical site necrosis expanded to approximately 12 × 5 mm in size. In the following week, ulceration was observed at the wound site, and it was considered difficult to continue conservative observation, prompting further debridement and initiation of negative pressure wound therapy (PICO system) for four weeks. After this course of treatment, the wound gradually healed approximately two and a half months after surgery.

## Discussion

We experienced a patient who developed MSFN following right mastectomy. Although this complication is commonly reported to occur in 5-30% of cases [2], several noteworthy aspects were present in this case. In addition to hypertension, an established independent risk factor for MSFN [3], the patient exhibited severe preoperative anxiety and encountered an exceptionally rare intraoperative event, the loss of a suture needle, which required approximately 60 minutes of surgical interruption and resulted in prolonged operative time. We speculate that the convergence of these three adverse factors, a triad of risks, likely contributed synergistically to the development of massive MSFN.

The incidence of intraoperative needle loss is generally low, estimated at approximately 0.06-0.1% in both open and minimally invasive surgeries; however, it is an event that can occur to any surgeon, and strict protocols for its management have not been fully established [11,12]. By contrast, retained surgical instruments (RSI) are regarded as “never events,” and rigorous protocols to prevent their occurrence have been implemented [13,14]. In our case, we adhered to these protocols by temporarily interrupting the procedure after the needle was lost and conducting a thorough search, including direct visual inspection and intraoperative radiography. As a result, the operative time was prolonged by approximately 60 minutes compared with the usual duration of less than two hours [10].

While preventing the retention of foreign bodies is of utmost priority, and our approach effectively eliminated the risk of RSI, prolonged operative time carries its own consequences. Extended anesthesia increases the risk of hypothermia, while prolonged exposure leads to desiccation and cooling of the surgical field, as well as sustained traction that may impair local perfusion, thereby exacerbating flap ischemia [15,16]. Indeed, the final operative time in this case was three hours and 24 minutes, substantially longer than the typical two-hour duration [10], and this extension likely had a considerable negative impact on flap viability. Although such intraoperative interruptions are unavoidable for patient safety [13,14], surgeons must remain mindful of the potential consequences, including compromised local tissue perfusion and increased risk of infection. Particularly for patients at high risk of MSFN, it is essential to establish clear guidelines that account for these additional considerations.

In this case, undetected preoperative hypertension may also have adversely affected microcirculation at the surgical site, underscoring the importance of adequate blood pressure control prior to surgery. Hypertension is recognized as an independent risk factor that significantly increases the likelihood of MSFN [3]. However, the patient had discontinued antihypertensive medication prescribed at another hospital on her own judgment, and she did not disclose a history of hypertension during the preoperative interview. As a result, this risk factor was not identified until the day before surgery, when her systolic blood pressure reached the 200 mmHg range, requiring intravenous nicardipine administration. Postoperatively, her blood pressure also transiently rose to the 160 mmHg range. Such marked fluctuations in blood pressure likely disrupted microcirculatory stability and created an unfavorable environment for wound healing [17].

Furthermore, in this case, the patient exhibited marked preoperative anxiety and stress responses, which cannot be overlooked. Psychological stress is known to alter autonomic, endocrine, and immune functions, thereby contributing to delayed wound healing [6]. In the present case, heightened preoperative anxiety may have activated the sympathetic nervous system, resulting in elevated blood pressure, and may also have influenced postoperative immune and inflammatory responses [18,19]. These observations suggest that comprehensive perioperative management, including attention to psychological well-being, can contribute to improved wound healing. Interventions such as fostering reassurance through thorough informed consent, administration of anxiolytic agents when appropriate, and the use of counseling have been reported to alleviate perioperative anxiety and improve postoperative outcomes [20]. Thus, rather than compartmentalizing physical and psychological factors, an integrative perspective encompassing the entire perioperative period is essential.

## Conclusions

In conclusion, this case was notable for the rare convergence of a known risk factor (hypertension), a psychological factor (preoperative anxiety), and an unforeseen intraoperative event (surgical interruption and prolonged operative time due to loss of a suture needle), likely contributed to extensive MSFN following a simple mastectomy. Future efforts should focus on expediting intraoperative search protocols and developing guidelines that balance surgical safety with physiological risks through integrated perioperative management addressing physical, psychological, and procedural factors.
